# Case report: Complete clinical remission of feline progressive histiocytosis after multimodal treatment including electrochemotherapy

**DOI:** 10.3389/fvets.2024.1397592

**Published:** 2024-08-22

**Authors:** Bruna Voltolin de Sena, Paula Baêta da Silva Rios Turquete, Pedro Antônio Bronhara Pimentel, Isabella Oliveira Almeida, Gleidice Eunice Lavalle, Karen Yumi Ribeiro Nakagaki, Antonio Giuliano, Paulo Ricardo de Oliveira Paes, Rodrigo dos Santos Horta

**Affiliations:** ^1^Department of Veterinary Medicine and Surgery, Veterinary School, Universidade Federal de Minas Gerais, Belo Horizonte, Minas Gerais, Brazil; ^2^Director and Technical Responsible at CELULAVET, Belo Horizonte, Brazil; ^3^Department of Veterinary Clinical Science, Jockey Club College of Veterinary Medicine, City University of Hong Kong, Hong Kong, Hong Kong SAR, China

**Keywords:** cat, histiocyte, Iba-1, electroporation, bleomycin

## Abstract

Feline histiocytic diseases are uncommon and rarely reported. Feline progressive histiocytosis (FPH) is the most common histiocytic disease in cats, predominantly affecting middle-aged animals. The most common presentation is the cutaneous form with solitary or multiple cutaneous nodules. A female, mixed-breed 6-year-old cat was presented with a 9-month history of a nodule in the nasal planum and was diagnosed by histopathology with histiocytic proliferation. At the time of diagnosis, new nodules were discovered on the lower lip, digit, and two lesions in the tail region, with the largest measuring 1.5 cm. Supplementary immunohistochemistry, showed immunolabeling for Iba-1 that in combination with the clinical course of the disease, confirmed the diagnosis of FPH. No response to chemotherapy treatment with lomustine alternated with doxorubicin was achieved. Toceranib phosphate resulted in a transient response and, stable disease for a short period (6 weeks). Electrochemotherapy with bleomycin was initiated and resulted in partial remission. Later on, chlorambucil was also started. Ultimately, the combination of all three treatments led to a complete response and disappearance of all the lesions. FPH is considered a disease resistant to various treatments, and effective treatments have not been reported. In this case report, we describe a successful multimodal therapeutic approach that resulted in complete resolution of the FPH and long-term survival (460 days without external lesions at the time of death). Further studies are necessary to confirm the efficacy of this therapeutic approach.

## Background

Histiocytes derive from CD34+ stem cell precursors, which differentiate into macrophages and dendritic cell lineage. The latter includes interstitial dendritic cells (ICs) located in local perivascular areas and various organs, as well as Langerhans cells (LCs) occurring within the epithelia of the skin, gastrointestinal, respiratory, and reproductive tracts (1). Dendritic cells are the most potent antigen-presenting cells for inducing immune responses in naive T cells. Faulty interaction between dendritic cells and T cells can lead to disordered immune regulation and contribute to the development of histiocytic disorders (2). Most histiocytic diseases in dogs and cats involve proliferations of IC but other lineages may be alternatively involved (3).

Histiocytic disorders are documented in humans, dogs, and cats; however, their etiology and pathogenesis remain unknown, and the course is typically fatal (4–6). In human medicine, there are many subtypes of histiocytic disorders. The latest publication by the Histiocyte Society classified histiocytoses based on radiological, histopathological, phenotypic, and molecular characteristics into the following groups: Langerhans cell-related (group L), cutaneous and mucocutaneous (group C), malignant histiocytosis (group M), Rosai-Dorfman disease (group R), hemophagocytic lymphohistiocytosis, and macrophage activation syndrome (group H) (7). In dogs, histiocytic manifestations include reactive histiocytosis, histiocytoma, cutaneous Langerhans cell histiocytosis, and more severe forms such as histiocytic sarcoma and hemophagocytic histiocytic sarcoma, all of which can be presented as localized and/or systemic disease (2).

Feline histiocytic diseases are uncommon and rarely reported. These disorders include histiocytic sarcoma (HS) (originating from ICs), hemophagocytic histiocytic sarcoma (originating from macrophages), feline progressive histiocytosis (FPH) (originating from ICs), pulmonary Langerhans cell histiocytosis, and, the sporadically reported, feline histiocytoma (originating from LCs) (6).

FPH is the most common histiocytic disease in cats, typically manifesting with the cutaneous form. Nevertheless it remains a rare disease which affects most females, typically middle-aged to elderly cats, without any breed predilection (8). The etiology of FPH is not well understood, but chronic antigenic stimulation has been considered a triggering factor in its development. Clinically, it behaves like a low-grade HS originating from resident dendritic cells within the skin (3). Initially, solitary or multiple non-pruritic and painless cutaneous nodules appear, mainly located on the head, lower extremities, and trunk. The surface is often alopecic and may become ulcerated (9). The lesions may increase in size, coalesce into plaques, or spontaneously decrease in size, but complete regression does not occur (3). Although initially confined to the skin, within a month to 3 years, they may progress to a malignant histiocytic neoplasm with metastases to lymph nodes, liver, spleen, kidneys, lungs, and bone marrow, ultimately culminating in spontaneous death or euthanasia. Conclusive diagnostic confirmation is based on histopathological, and immunohistochemical evaluations in combination with the clinical presentation (3, 9).

Histologically, the lesions consist of diffuse and dense infiltrates of histiocytic cells in the dermis and subcutaneous tissue, poorly demarcated (3, 10). In the early stages, they are well-differentiated with a low mitotic count, but with progression, cellular pleomorphism, increased mitotic count, and multinucleation may occur, resembling HS (9). Distinguishing between histiocytic subtypes is not possible with routine histopathology and requires immunohistochemistry. Among histiocytic disorders, the immunophenotypic profile for hemophagocytic histiocytic sarcoma is IBA-1+, CD204+, MHC-II-, E-cadherin-; for HS, Reactive histiocytosis and FPH, it is IBA-1+, CD204±, MHC-II+, E-cadherin-; and in histiocytoma and cutaneous Langerhans cell histiocytosis (including feline pulmonary form) it is IBA-1+, CD204±, MHC-II+, E-cadherin+. In FPH variations have been observed such as CD5+ and E-cadherin+ in 50% and 10% of cases, respectively (2, 9, 11).

Addressing histiocytic neoplasia presents challenges not only in diagnosis but also in treatment. Therapies with corticosteroids, antibiotics, interferon, L-asparaginase, nitrogen mustard, vincristine, and vinblastine have been described but have not shown satisfactory results (3, 12). Surgical resection is questionable once local or distant recurrence is common (2). However, patients at the initial presentation with no signs of metastasis (such as lymph node enlargement) can benefit from wide surgical resection showing increased disease-free interval (10). While treatment in human medicine is standardized, based on the type of histiocytosis, disease extent, phenotype, and molecular characteristics, there are not effective treatment or standard of care for feline histoicytic disease. For example, in common histiocytoses in adults, such as Langerhans cell histiocytosis, Erdheim-Chester disease, and Rosai-Dorfman disease, when they present MAPK/ERK pathway mutations, the treatment includes cobimetinib/trametinib. However, a range of drugs such as prednisolone, vinblastine, hydroxyurea, thalidomide, cytarabine, methotrexate, and interferon alfa-2a are used, tailored to the specific disease (7).

Electrochemotherapy (ECT) is a local treatment that combines the action of a cytotoxic drug with electroporation of the cell membrane through electrical pulses, allowing greater penetration and enhancement of hydrophilic molecules such as bleomycin or cisplatin. It has been used as a single or adjuvant therapy for various types of superficial cutaneous solitary tumors showing high response rates with minimal side effects, and remarkable responses. However there is a variability in response rate depending on the tumor types. Tumors showing a high response rate especially for small masses are: oral canine melanoma, (around 90%), feline cutaneous squamous cell carcinoma (around 80%), equine sarcoids (around 97%), around 100% for tumors smaller than 2 cm^3^ (13, 14).

This study had the objective to report the first case of FPH with documented complete response of the cutaneous lesions using a multimodal therapeutic approach, including ECT.

## Case description

**Figure 1:**
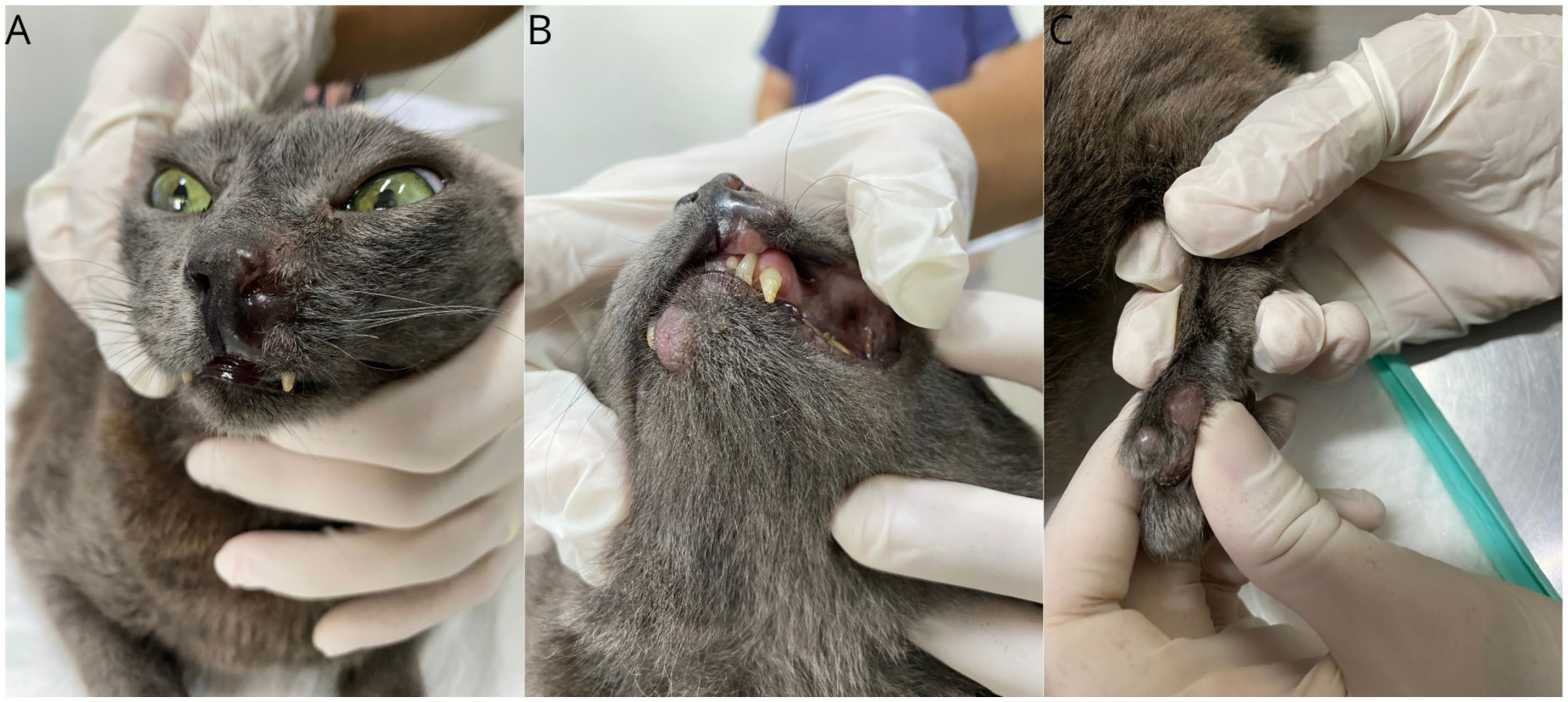
Gross features of FPH skin nodules. Presence of nodular lesions in the muzzle area **(A)**, lower and upper lip **(B)**, and on the digit of the left hind limb **(C)**.

A 6-year-old spayed female mixed-breed domestic cat, weighing 3.6 kg, was referred with a history of a nodular lesion on the nasal planum that had persisted for the past 9 months. On physical examination, vital parameters were within normal range and the cat was otherwise healthy except for the presence of nodular, alopecic, non-ulcerated lesions in the nasal region, lower lip, digit, and two lesions on the tail, with the largest measuring 1.5 cm (Figure 1). The patient tested negative on the SNAP Combo FIV/FeLV (Alere^®^) and an incisional biopsy performed at another veterinary facility was diagnostic of histiocytic proliferative process. The biopsy revealed a marked presence of histiocytic infiltrates in the superficial and deep dermis. The cells had broad, slightly eosinophilic cytoplasm, and central, large, and rounded nuclei, occasionally showing slight indentation, margined chromatin, and evident nucleoli. There were 12 mitotic figures in 2.37 mm^2^, moderate pleomorphism with occasional karyomegaly, and a discreet lymphoplasmacytic infiltrate.

**Figure 2:**
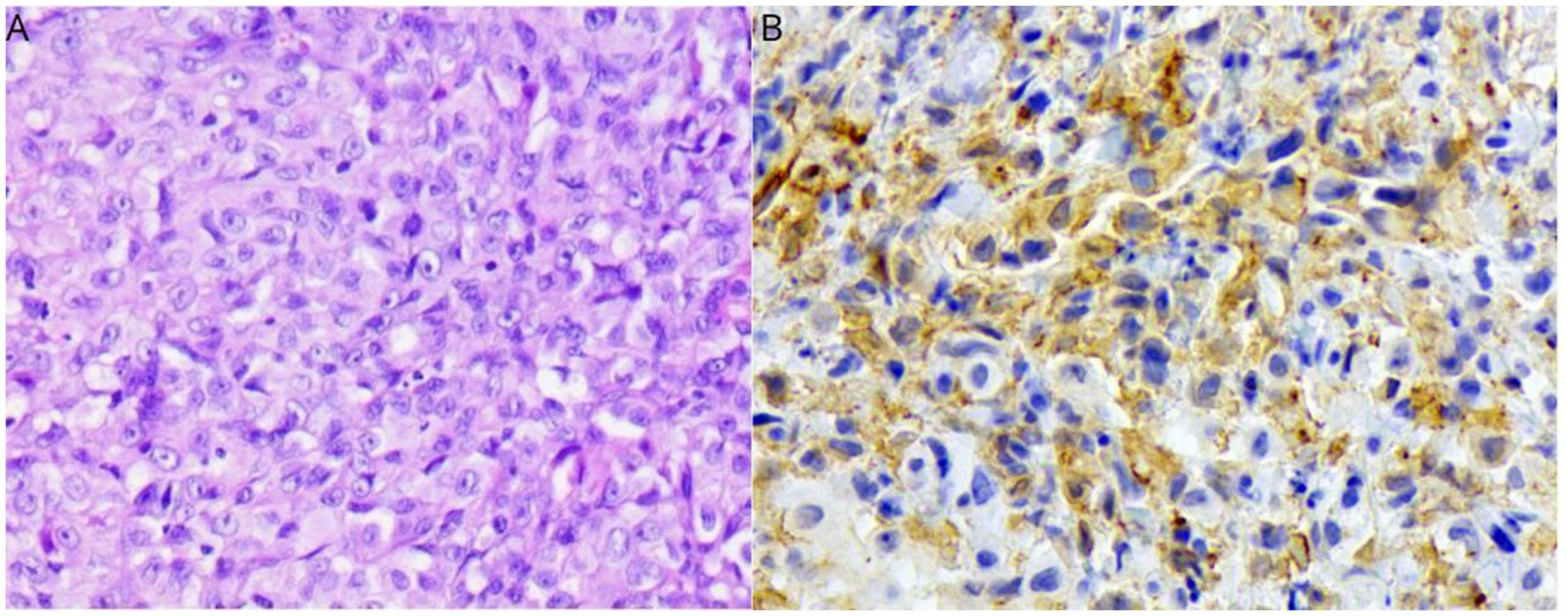
Histological features of FPH from the nasal planum nodule. H&E (40× objective) **(A)**; Positive immunolabeling for Iba-1 **(B)** (40× objective).

An immunohistochemistry panel was requested on the previously collected sample including Iba-1, CD3, Granzyme B, CD20, CD117, MUM1, Pax-5, and Pan cytokeratin markers. Only Iba-1 was positive, confirming the histiocytic origin (Figure 2). The procedure included 4 μm sections, deparaffinized in xylene and hydrated in decreasing concentrations of ethanol, followed by rinsing in distilled water. For antigen retrieval, a high pH solution (Target Retrieval Solution High pH-DM828, K800221-2 EnV FLEX+, High pH Link, DAKO) was used in a pressure cooker (PascalR, Dako), except for CD20 (Polyclonal, Thermo Fisher Scientific) which did not undergo antigen retrieval. The slides were then cooled at room temperature for 20 min and rinsed with deionized water. To block peroxidase, the slides were immersed in ready-to-use hydrogen peroxide (EnVisionTM FLEX PEROXIDASE-BLOCKING REAGENT SM801, K800221-2 EnV FLEX+, High pH Link, DAKO). The sections were then washed with tris solution (pH 7.4) and subsequently blocked for nonspecific protein reactions (serum-free protein block– DAKO, X0909). This was followed by incubation with the primary antibody for 18 h at 4°C. Finally, amplification and detection were performed with EnVision FLEX/HRP, SM802 (Dako) and the chromogen diaminobenzidine (EnVision FLEX DAB+CHROMOGEN, DM827, DAKO), followed by counterstaining with Harris hematoxylin. Additional information can be found in Table 1. Additional investigation and overall assessment included abdominal ultrasound, three-view thoracic radiography, complete blood count, and serum biochemistry (Urea, Creatinine, ALT, AST, GGT, alkaline phosphatase, proteins and fractions, calcium, and phosphorus), but they were all unremarkable.

**Table 1:** Primary antibodies used for immunohistochemical analysis to characterize the neoplastic cells in feline progressive histiocytosis.

Antibody	Species	Clone	Dilution	Antibody manufacturer
Iba1	Rabbit	Polyclonal	1:1000	Wako
CD3	Rabbit	Polyclonal	1:200	Dako/Agilent
Granzyme B	Rabbit	Polyclonal	1:50	Cell Marque
CD20	Rabbit	Polyclonal	1:500	ThermoFisher Scientific
CD117	Rabbit	Polyclonal	1:200	Dako/Agilent
MUM1	Rabbit	BC5	1:250	Biocare Medical
Pax-5	Mouse	Dak-Pax5	Predilute	Dako/Agilent
CK Pan	Mouse	AE1AE3	Predilute	Dako/Agilent

**Figure 3:**
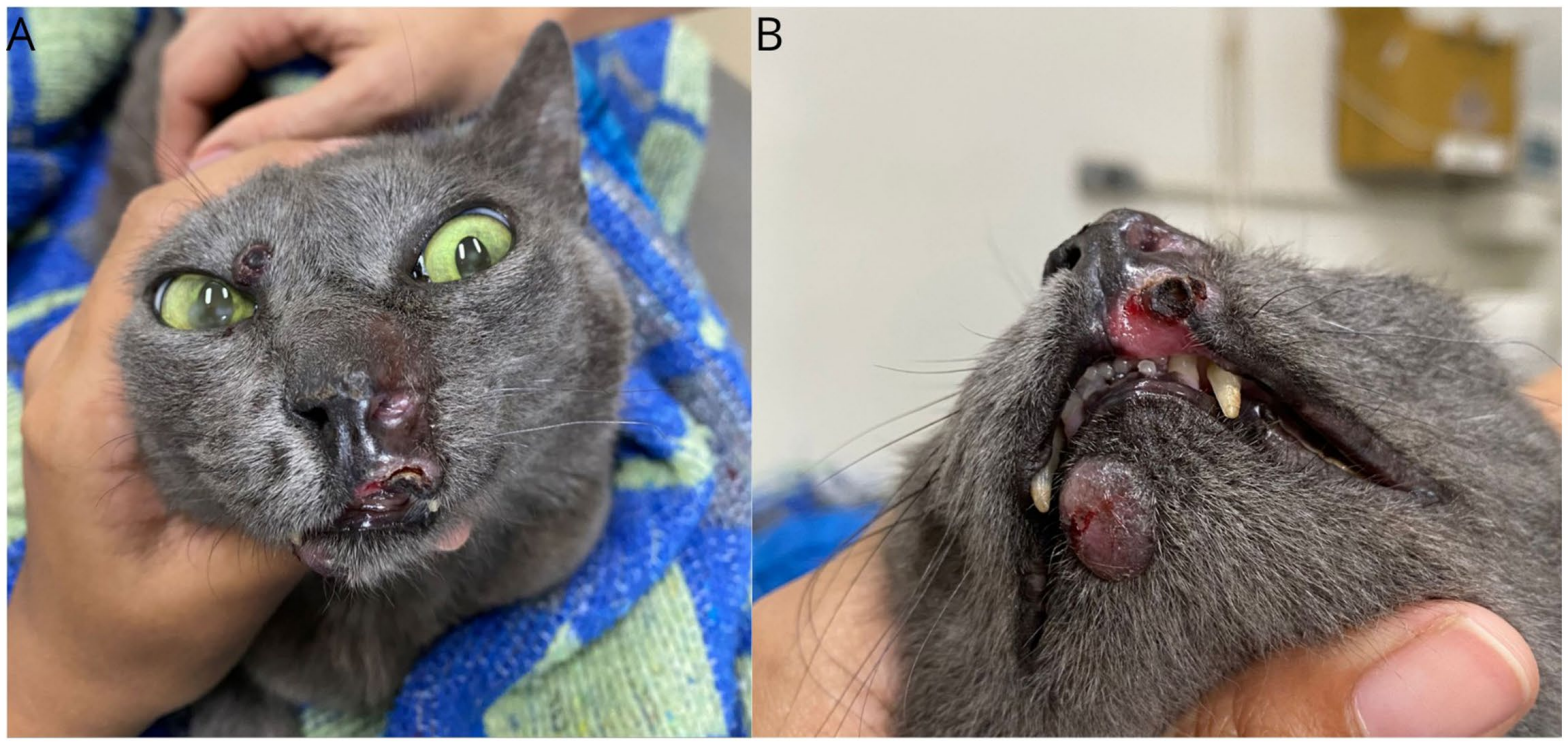
Gross features of FPH skin nodules. Clinical presentation after the first cycle of lomustine and doxorubicin with a 14-day interval. Lesions showing slight increase in size **(A)** and ulceration with the presence of crust on the upper lip **(B)**.

Following the diagnosis of FPH, chemotherapy treatment was initiated with oral lomustine (Compounding pharmacy, Brazil) at a dose of 35 mg/m^2^ and intravenous doxorubicin (Rubidox^®^-Bergamo, Brazil) at a dose of 1 mg/kg, alternated with a 14-day interval between medications. Prednisolone (Prediderm^®^-Ourofino, Brazil-0.7 mg/kg, PO, BID) and omega-3 (Omega TOP 3^®^-Agener União, Brazil-500 mg, PO, SID, continuous use) were also prescribed. There was no objective response 14 days after a single dose of each chemotherapeutic agent, and the disease progressed, with increasing size and number of lesions, some nodules also became ulcerated (Figure 3). In the same visit, grade III neutropenia (630/μL, reference 3.000–11.000) and grade I thrombocytopenia (126,000/μL, reference 175,000–500,000) were observed, resulting in a single subcutaneous dose of filgrastim (5 mcg/kg). The treatment was changed, prednisolone, lomustine and doxorrubicin were suspended and toceranib phosphate was started at a dose of 10 mg/cat on Mondays, Wednesdays, and Fridays, while maintaining omega-3 supplementation.

**Figure 4:**
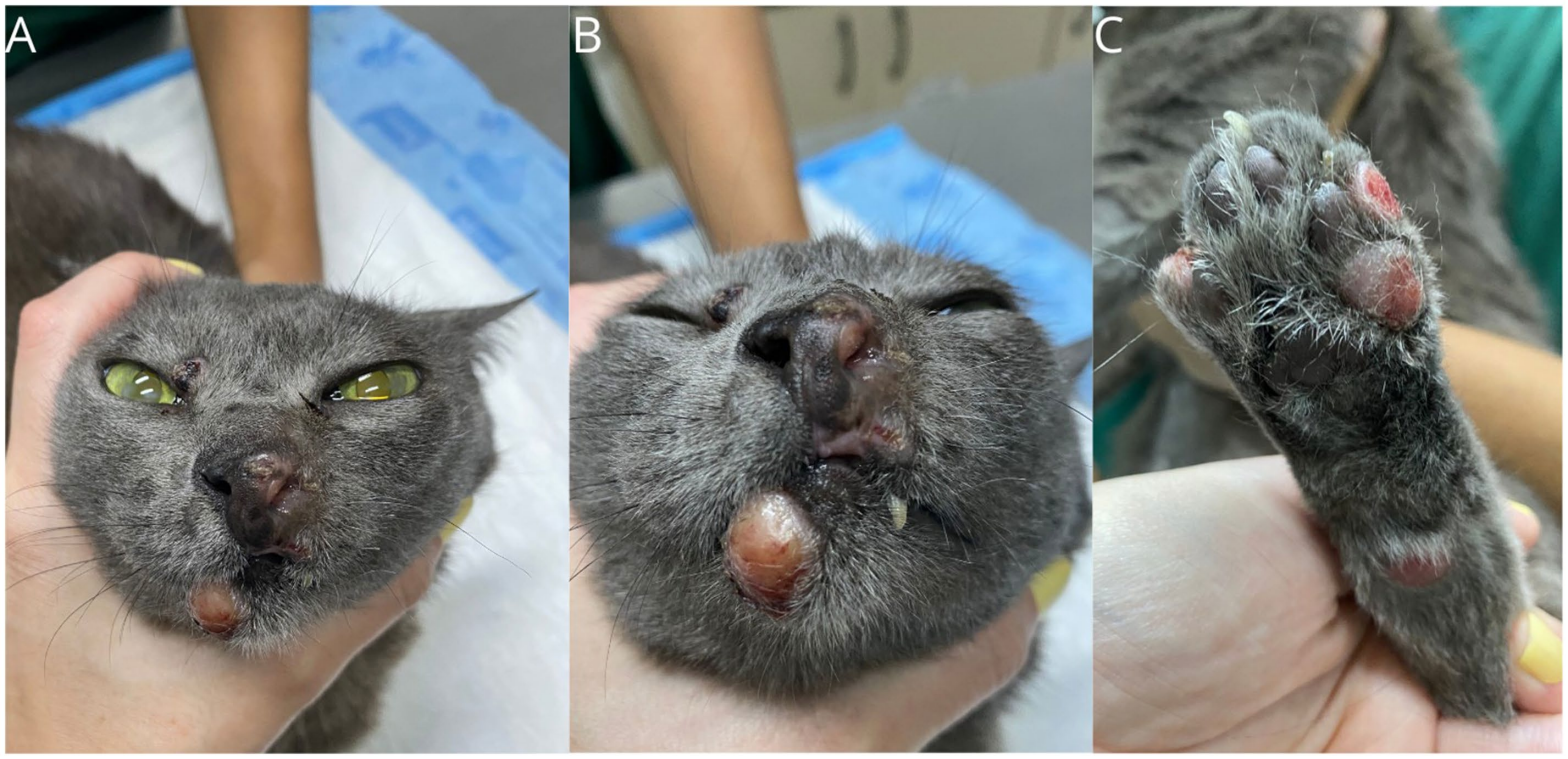
Gross features of FPH skin nodules. Clinical presentation after 21 days of starting toceranib phosphate, demonstrating pale and dry lesions. Lesions on the muzzle **(A)**, lips **(B)**, and digit **(C)**.

After 21 days of toceranib phosphate use, the patient was reassessed, and the lesions appeared of similar size but less edematous, erythematous and exsudative (Figure 4). Blood tests, including complete blood count and serum biochemistry, were repeated and found to be within normal values. The treatment was continued, followed by a new attempt with a single oral dose of lomustine (30 mg/m^2^) 33 days after initiantig toceranib. This decision was made due to the potential sensitization of lomustine by a tyrosine kinase inhibitor, although there is no description of such benefit for FPH (15).

**Figure 5:**
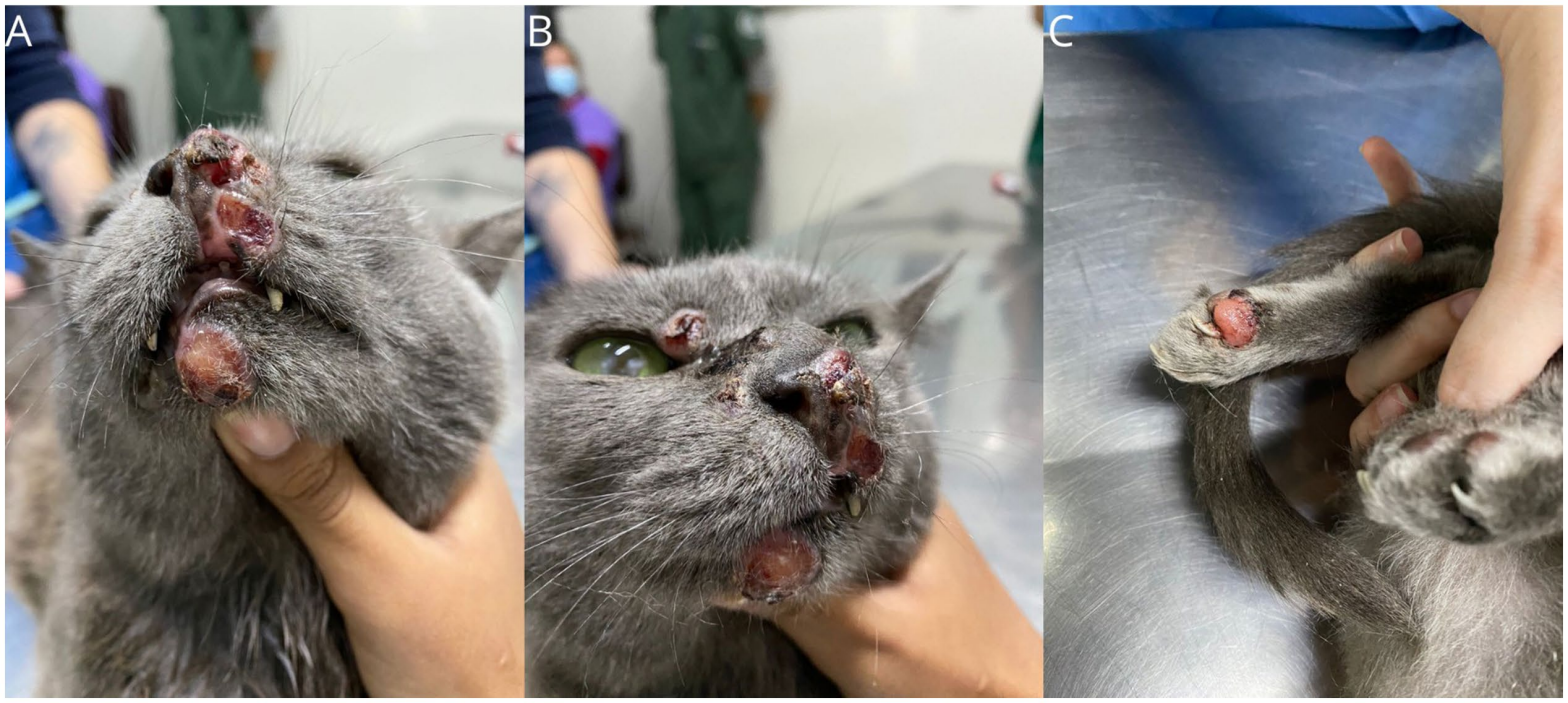
Gross features of FPH skin nodules. Clinical presentation after a short term stable disease with toceranib, showing an increase in size and ulceration of the lesions. Lesions on the muzzle **(A, B)**, lips **(A, B)**, right upper eyelid **(B)**, and digit **(C)**.

**Figure 6:**
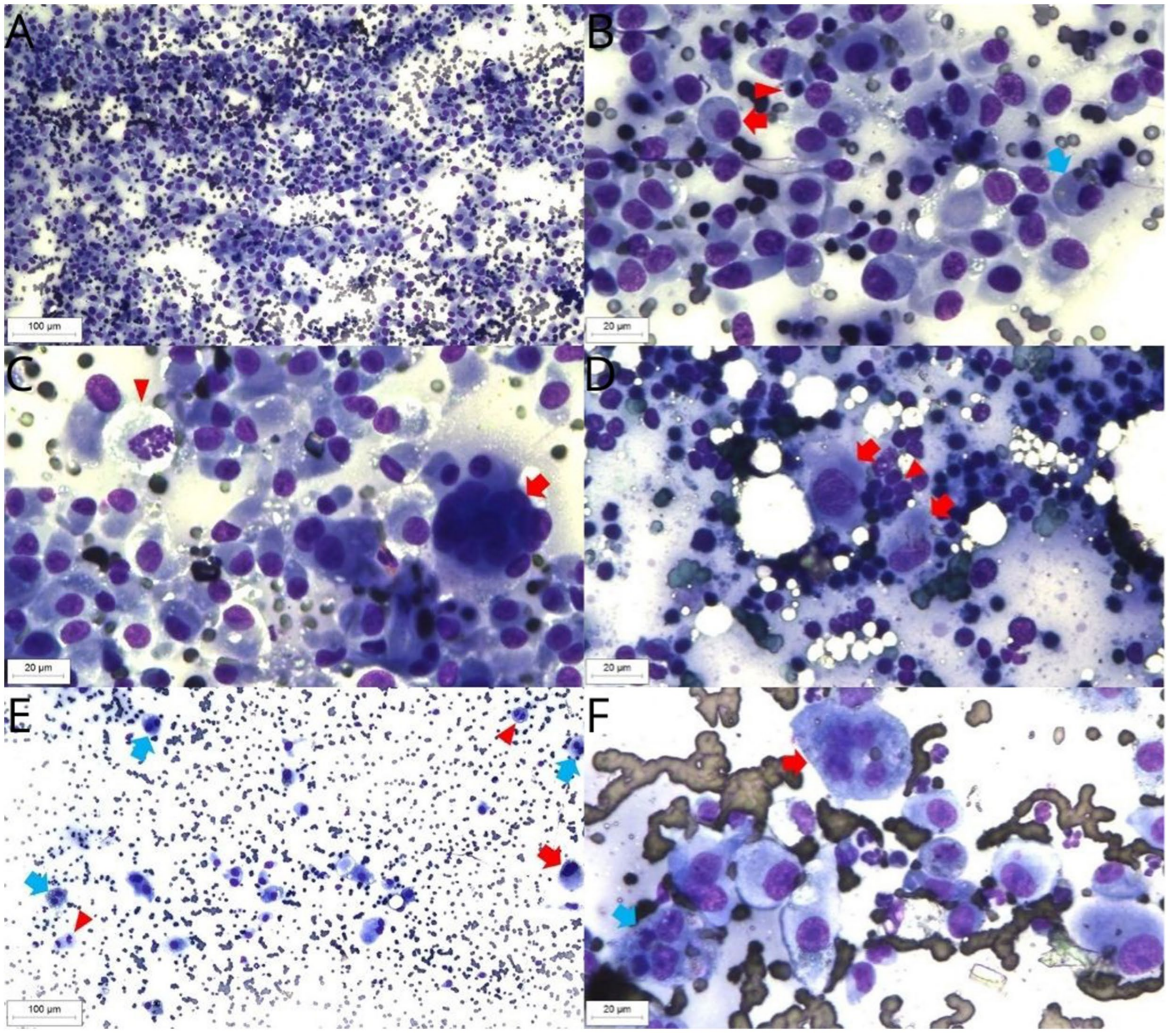
FPH cytology, Diff Quick staining. Retropharyngeal lymph node sample. Background with moderate red blood cells numbers. High large histiocytic cells, moderate small lymphocytes and low neutrophils numbers (10× objective) **(A)**. Retropharyngeal lymph node sample. Histiocytic cells have a moderate nucleus-to-cytoplasm ratio, rounded or oval nucleus, moderate anisokaryosis, single nucleolus and low to moderate basophilic cytoplasm (red arrow). Presence of cytophagocytosis (blue arrow). The small lymphocytes present typical morphology (arrowhead) (40× objective) **(B)**. Retropharyngeal lymph node. Multinucleated histiocytic cell (arrow) and mitotic figure (arrowhead) (40× objective) **(C)**. Popliteal lymph node sample. Presence of high small lymphocytes (arrowhead) and a low histiocytic cells numbers, with the last ones exhibiting the same pattern observed in the retropharyngeal lymph node sample (red arrow) (40× objective) **(D)**. Superficial cervical lymph node. Background with high red blood cells numbers. Presence of large histiocytic cells, showing high anisokaryosis (red arrow), binucleated cells (blue arrow) and mitotic figures (arrowhead) (10× objective) **(E)**. Superficial cervical lymph node sample. Histiocytic cells with a prominent single nucleolus, binucleation with cytophagocytosis (blue arrow) and multinucleation (red arrow) (40× objective) **(F)**.

At the subsequent follow-up, after broadly 6 weeks of stable disease there was a significant progression of the lesions in the nasal region, along with ulceration of the cutaneous lesions (Figure 5) and an increase in size of the left popliteal lymph node. The lymph node underwent fine-needle aspiration cytology (FNAC), confirming the FPH involvement of the popliteal lymph node (Figure 6D). The owner reported that the animal was experiencing itching and pain in the limb lesions due to excessive licking, along with lethargy and hyporexia. Supportive treatment was also prescribed, including gabapentin (Compounding pharmacy, Brazil – 6 mg/kg, BID, continuous use), mirtazapine (Mirtz^®^ – Agener União, Brazil-2 mg/cat, every 48 h, for 14 days) and meloxicam (Flamavet^®^ – Agener União, Brazil-0.02 mg/kg, SID, for 14 days). Due to the lack of success with the instituted treatments, electrochemotherapy (ECT) was performed with eight square wave pulses, each lasting 100 microseconds, with an amplitude of 1,000–1,300 V/cm, and a frequency of 5 kHz. Bleomycin (Bonar^®^ – IMA S.A.I.C., Argentina) was administered intravenously at a dose of 15 IU/m^2^, 8 minutes prior to electroporation. Electroporation was performed on all lesions, with a 1–2 cm margin around each lesion.The first 4 applications were performed using the VETCP 125 electroporator device, which was later substituted for the ePORE, from the same manufacturer, using it in the last two applications. The anesthetic protocol used for the ECT sessions included methadone (0.3 mg/kg IM) combined with dexmedetomidine (5 mg/kg IM) (Dexdomitor^®^ – Zoetis, Brazil) and ketamine (1 mg/kg IM) (Cetamin^®^ – Syntec, Brazil) as pre-anesthetic medication; propofol (2 mg/kg IV) (Propovan^®^ – Cristália, Brazil), ketamine (1 mg/kg IV) (Cetamin^®^ – Syntec, Brazil), and fentanyl (2.5 μg/kg IV) (Fentanest^®^ – Cristália, Brazil) for anesthetic induction. Sevoflurane was used as the inhalation anesthetic for maintenance, along with fentanyl (5 μg/kg IV) (Fentanest^®^ – Cristália, Brazil).

**Figure 7:**
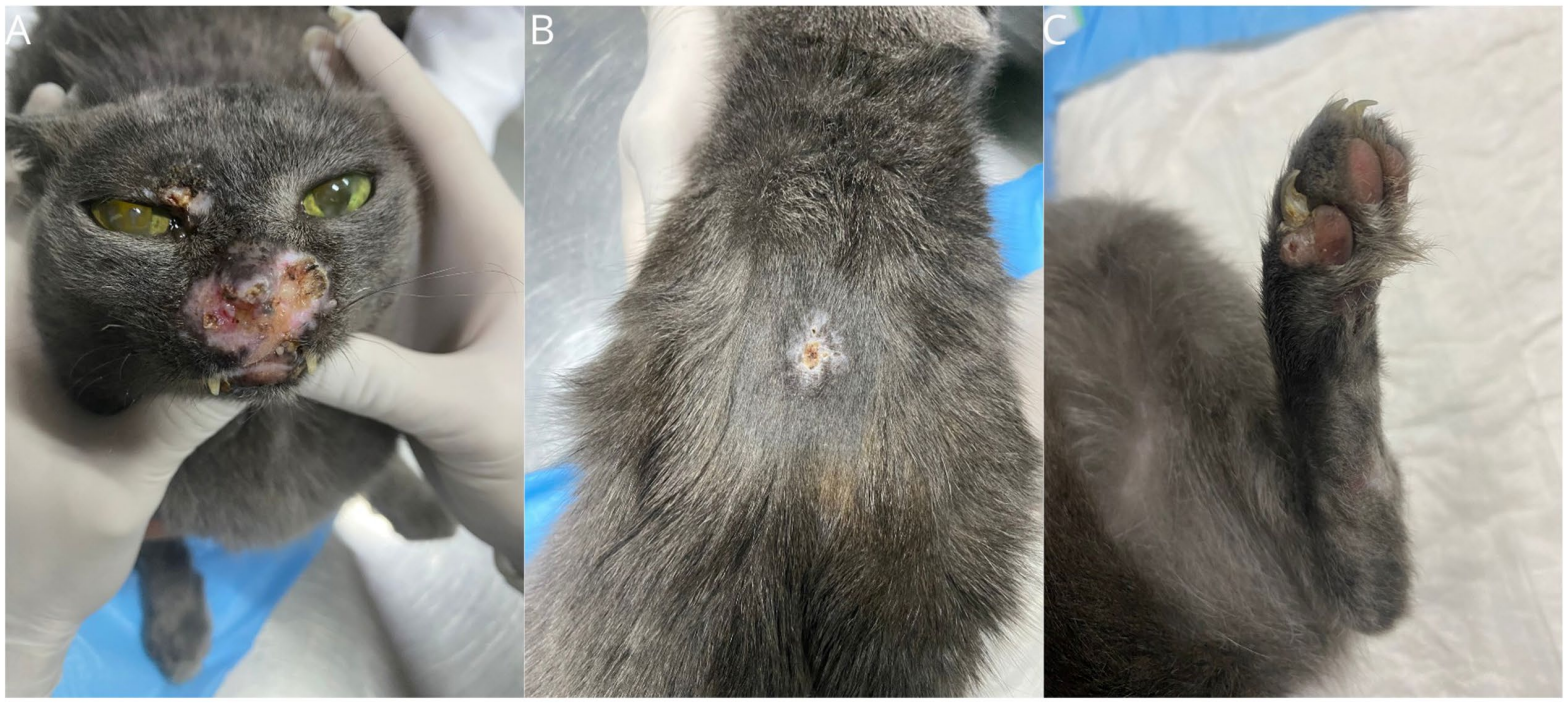
Gross features of FPH skin nodules. Clinical presentation 18 days after the third ECT session. Presence of crusts between the nasal planum and upper lip and vibrissal region **(A)**, dorsum **(B)**, and fourth digit **(C)**, sites of ECT application in the healing process.

A total of six sessions were conducted with intervals of 22, 22, 32, 50 and 134 days, respectively (median of 32 days). Hematological and serum biochemistry pre-anesthetic exams were within normal limits before each treatment. From the first session a partial response was obtained followed by a progressive reduction of size of the nodules. After 18 days from the third session, there was 80% of reduction in size of the nodules (Figure 7). Thoracic radiographs and abdominal ultrasound were performed during follow-up, and there were no signs of metastasis.

After 21 days from the fourth ECT session, new lesions were observed during the physical examination, including the upper and lower eyelids, inter-scapular dorsal region, and recurrence in the nasal region, left hind limb digital pad, tarsal region of the right hind limb, and two nodules on the tail. Additionally, there was another nodule measuring 3 cm, firm, adherent, and deep in the central cervical region. Cervical ultrasound suspected an enlargement on the left thyroid gland or left medial retropharyngeal lymph node, and guided fine-needle aspiration cytology was performed for evaluation. The cytology described the abundant presence of histiocytes, oval to spindle-shaped, with low to variable nucleus-to-cytoplasm ratio, oval nuclei, intense anisocytosis, coarse chromatin pattern, single and inconspicuous nucleoli, discretely basophilic and poorly demarcated cytoplasm, mild to moderate presence of binucleated cells and large cells, as well as cytophagocytosis and erythrophagocytosis, with more than 5 mitotic figures per field (Figures 6A–C). The patient was referred for computed tomography, revealing enlargement of medial retropharyngeal lymph node (3.5 cm × 1.8 cm × 1.8 cm), right medial retropharyngeal lymph node (2.1 cm × 0.8 cm × 0.3 cm), and left superficial cervical lymph node with a slight increase in volume, as well as the left mandibular lymph node with a moderate increase in volume. There was no abnormalities on the thyroid glands. In light of these findings, treatment was intensified with the addition of chlorambucil at a dose of 2 mg/cat on Mondays, Wednesdays, and Fridays.

The patient was assessed 21 days after the combination therapy with toceranib phosphate and chlorambucil. The retropharyngeal lymph node was no longer palpable and the cutaneous lesions were smaller.

**Figure 8:**
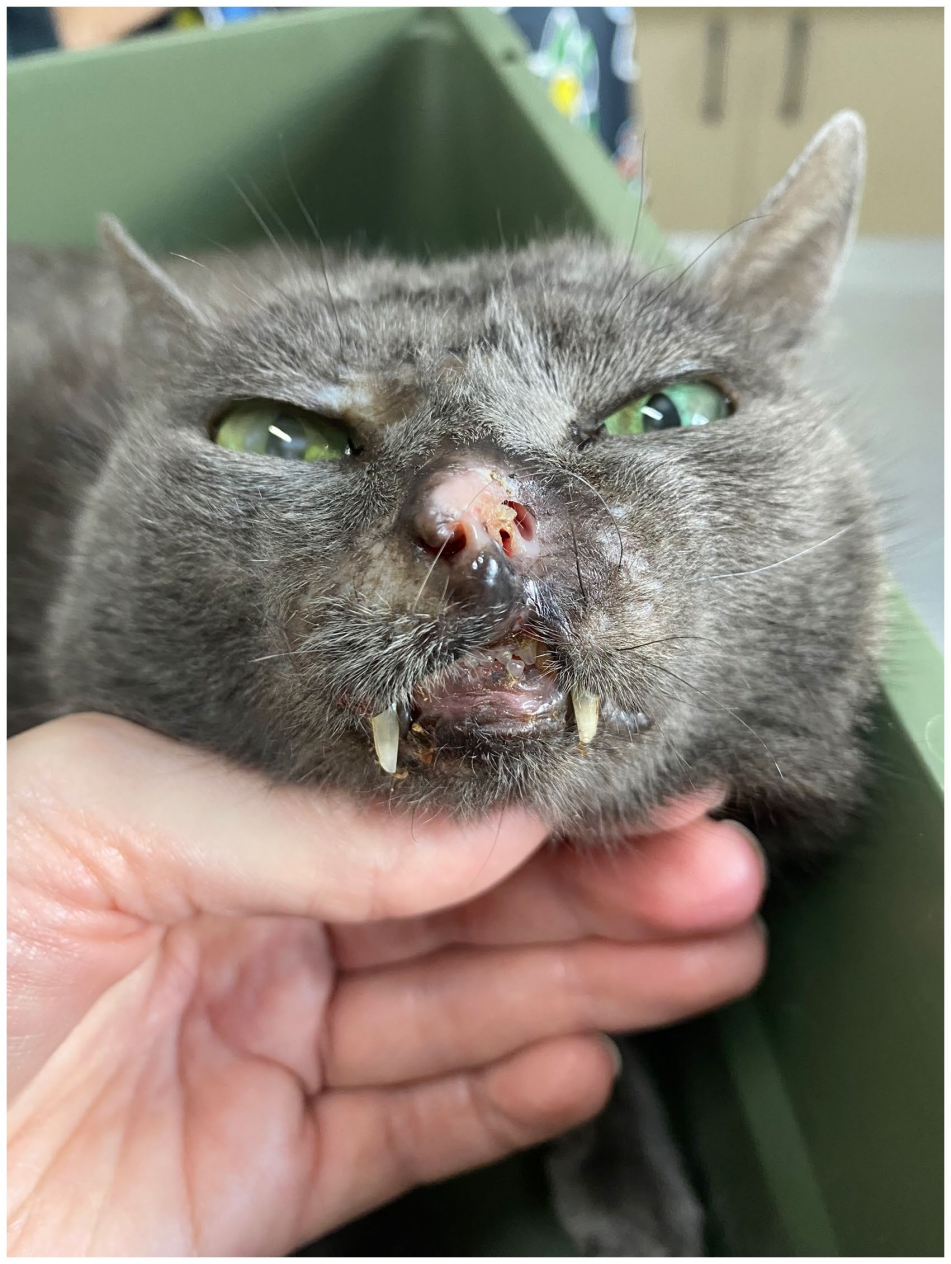
Gross features of FPH skin nodules. Patient in complete remission with treatment involving toceranib phosphate, chlorambucil, and 3 months after the sixth ECT session. Scar on the nose with exposure of nasal mucosa.

To control the remaining skin lesions, ECT treatment was continued. However, a longer interval between sessions was instituted in order to reduce the costs and improve patients welfare. After completing 6 ECT sessions, 200 days from the start of toceranib phosphate, and 114 days from the combination of toceranib phosphate and chlorambucil, there was complete remission of the cutaneos lesions observed (Figure 8) and there was no abnormalities on physical examination, abdominal ultrasound and thoracic radiographs. Lethargy and hyporexia was noted up to 2 days after ECT during sessions 1, 2, 4 and 6. Additionally, the patient exhibited respiratory discomfort following crust formation in the nasal region after the first two sessions, with immediate improvement observed after crust removal. Asymptomatic grade I neutropenia was observed and monitored during continuous combined therapy.

Three months after the last ECT session, the patient presented with symptoms of hyporexia, lethargy and weight loss. An increase in the volume of the right superficial cervical lymph node was observed, and a sample was collected through fine-needle aspiration cytology. The cytology results showed increased cellular atypia compared to the previous examination. In addition to previously observed characteristics, round nuclei, prominent, single, and occasionally multiple nucleoli were noted. Multivacuolated nuclei, figures of cytophagocytosis and multinucleation, as well as atypical mitotic figures, were observed, favoring a suggestive diagnosis of HS (Figures 6E,F). Abdominal ultrasound, three-view thoracic radiographs, and blood tests were requested, but the animal passed away at home, most likley due to disease progression, 460 days following diagnosis. Post-mortem examination was declined by the owner.

## Discussion

As both FPH and HS originate from ICs, they are immunophenotypically identical and the differentiation between these two conditions lies in the more marked cellular atypia in HS compared to FPH (9). Nevertheless, the typical clinical appearence of FPH and HS are different, which can aid in diagnosis. FPH initially has an indolent behavior, usually confined to single or multiple cutaneous nodular lesions while HS commonly exhibits rapid dissemination to multiple organs with lesions in the spleen, lymph nodes, lungs, and skin (16). As previously described, in our case report FPH showed a slow progression, starting with a single nodule on the face, followed by the development of multiple nodules throughout the body and involvement of the regional lymph nodes. The differentiation of FPH from HS in our case was straight forward considering the diagnosis at earlier stage. However, in the advanced stages of FPH, it could be clinically and histologically challenging to differentiate FPH from HS.

In a study involving 5 cats with immunohistochemical confirmed FPH, microscopic features on histopathology included large, round to polygonal neoplastic cells with a low nucleus-to-cytoplasm ratio. Additionally, the cytoplasm was mildly eosinophilic, and occasionally, neoplastic cells exhibited numerous cytoplasmic vacuoles and occasional binucleation. The nuclei were round to oval, often with a small single nucleolus, and 0–3 mitotic figures were observed per field (9). This description closely resembles the findings in the present case, except for the absence of vacuoles and binucleation.

During the course of the disease at least, three lymph nodes were affected, and histiocytic infiltration was confirmed by cytological examination. It was noted that cellular atypia worsened as the disease progressed in time and the last lymph node sampled exhibited the highest amount of atypia in cytological evaluation (10).

The immunohistochemical technique is recommended for confirming the histiocytic nature. To exclude differential diagnoses and establish a definitive diagnosis, immunohistochemical examination of the tumor sample was performed. There was no immunoreactivity for CD117, MUM1, CD3, CD20, ruling out diagnoses of mast cell tumor, plasmacytoma, T and B cell lymphomas, respectively. Positive immunolabeling for Iba-1, along with histomorphological characteristics and clinical progression, enabled the diagnosis of FPH. Immunostaining with Iba-1 has proven effective for the identification of histiocytes in cats, although it does not allow the differentiation of histiocyte subtypes (11, 17).

There is no robust data in the literature regarding the treatment of FPH. A study reported the use of corticosteroids, cyclosporine A, interferon-gamma, vincristine, vinblastine, and L-asparaginase in isolated cases, but the detailed clinical response of each drug individually was not described. Overall, patients did not respond well to these treatments (3). Another research study described a case of FPH treated with steroids that remained disease-free for 473 days with a follow-up of 665 days, and another case that used lomustine and metronomic cyclophosphamide with a 365-day follow-up, but the response of skin lesions to therapies was not detailed (8). Information on the response to lomustine in FPH is scarce; in the present study, disease progression was observed despite lomustine treatment. Doxorubicin also did not show benefits for the patient, and its use in FPH cases is not documented in the literature.

The pathogenesis of FPH is not well understood, making therapeutic choices challenging. The outcome of this case suggests the need for a multimodal therapy approach to target various pathways and potentially even stimulate an immune response (2). Growth factors influencing dendritic cells include fms-like tyrosine kinase 3 (FLT3), granulocyte-macrophage colony-stimulating factor (GM-CSF), tumor necrosis factor-alpha (TNF-α), interleukin 4 (IL-4), transforming growth factor beta 1 (TGF-β1), and stem cell factor (SCF) (18).

A study evaluated potential therapeutic targets, platelet-derived growth factor receptor beta (PDGFR𝛽), and KIT, in samples from 15 cats with histiocytic disorders, including 5 diagnosed with FPH. Immunostaining for PDGFR𝛽 was observed in 4 of these cats, while KIT was not expressed. Three FPH patients underwent tyrosine kinase inhibitor (TKI) treatment (masitinib or toceranib), with the one with the longest survival (223 days) showing weak PDGFR𝛽 immunostaining, while the other two cases who exhibited strong PDGFR𝛽 immunostaining survived only 110 and 45 days (8). The controversial result could be caused by the small number of cases and by including early and later stage disease with very different prognosis PDGFR𝛽 or others TK could be a potential target for this disease and toceranib could be a potential treatments for histiocytic disorders in cats. Toceranib phosphate targets PDGFR, as well as other receptors like FLT3 and vascular endothelial growth factor receptor (VEGFR). However, these additional targets have not been thoroughly investigated in the context of FPH. The use of toceranib in the patient in this case report resulted in stable disease for only 6 weeks.

Regulatory T lymphocytes (Treg) are involved in tumor-induced immune suppression and can be targeted by toceranib. In a study in dogs with various malignant tumors, there was a significant and selective reduction in patients’ Treg after 14 days of treatment with toceranib, which was synergistic with metronomic chemotherapy. This result demonstrates the potential immunomodulation of TKIs and metronomic chemotherapy in oncology patients, another mechanism that may have occurred in the patient in this case report; however, more studies are needed to understand the role of TKIs in cancer-induced immune suppression (19).

There is also evidence of synergism between TKI and ECT. A study evaluated the cytotoxicity of this combination in a human pancreatic carcinoma cell line with both bleomycin and cisplatin. Through the analysis of the cell death process, a synergistic effect was detected between sunitinib, a TKI similar to toceranib, and ECT with bleomycin (20). In the present report, it was observed a good response to treatment combining TKI, metronomic chemotherapy and ECT with a significant progressive reduction in lesions until complete remission was achieved.

The effectiveness of ECT in veterinary medicine is well established, and it is currently recommended as a neoadjuvant and adjuvant treatment for various solid tumors (13). In addition to direct cytotoxic effects, ECT can also trigger indirect antitumor effects by activating the immune system through the release of tumoral antigens and immunogenic cell death (ICD). It represents any form of cell death that effectively stimulates an adaptive immune response against antigens released by dying cells and releases substances that promote phagocytosis by dendritic cells and macrophages (21). Bleomycin administered intravenously results in a lower concentration of bleomycin within tumor cells, in comparison to intratumoral administration, which results in progressive damage to DNA at every cell cycle, until a catastrophic mitosis occurs, leading to cell death, similar to necrosis, which is a highly ICD. At higher concentrations or intratumoral injections, pseudoapoptosis may occur, which is less immunogenic (22, 23). Studies with cancer models in immunocompetent and immunocompromised mice have demonstrated variation in the efficacy of ECT, emphasizing the importance of the immune system. It was shown that immune activation induced by ECT in immunocompetent mice was essential for an efficient response against the tumor, while immunodeficient mice exhibited considerably reduced or absent responses to treatment (24).

Chlorambucil is a widely used alkylating agent in feline medicine, associated with a low incidence of adverse effects, and is well-tolerated, even when combined with other drugs such as corticosteroids. Chlorambucil is also used as an immunosuppressant and immunomodulatory drug, and could be beneficial in disease sustained by excessive immune function, like FPH (25). A study identified chlorambucil as a key chemotherapeutic agent that depletes PD-L1 in tumor cells. In an ovarian carcinoma cell line, chlorambucil was observed to mediate a 5-fold reduction in PD-L1 through transcriptional or post-transductional mechanisms, including the promotion of PD-L1 ubiquitination mediated by the GSK3β/β-TRCP signaling pathway. Furthermore, they demonstrated that depleting PD-L1 from neoplastic cells was responsible for inhibiting malignant cell growth (26). Information regarding PD-L1 in feline cancer is limited; however, a study in cats found PD-L1 expression in 100% of Feline SCC samples, and 80% positivity in fibrosarcoma (27). Nevertheless, for a better understanding of the interaction of chlorambucil’s effect on the tumor microenvironment, it would be necessary to include markers for PD-L1, Treg lymphocytes (FOXP3), and CD8 T lymphocytes, which was not performed.

In our case report, the FPH did not respond to maximum tolerated dose of chemotherapy with lomustine or doxorubicin, or to the tyrosine kinase inhibitor toceranib phosphate. However objective response was shown with the use of electrochemotherapy and then with the addition of chlorambucil, while still on the use of toceranib. A delayed immune response could be, at least partially, involved on the clinical response and long survival observed. However, further studies are necessary to understand the role of these therapeutic options in FPH.

The literature reports only 30 cases of FPH, with just 10 undergoing oncological follow-up for periods ranging from 1 month to 3 years. These cases show variable survival times, with a median survival of 407 days. In the present case, the patient survived approximately 2 months longer, maintaining a good quality of life, which is crucial in this disease, as the majority of cases often are euthanased due to lack of response and poor quality of life (3).

In conclusion, FPH proves resistant to various treatments, but the implementation of a multimodal therapy aiming at restoring anti-tumor immunity may enhance the chances of a response. ECT could be a useful tool for controlling FPH cutaneous lesions, which can significantly impact the patient’s quality of life. Repeated use of ECT for the management of cutaneous lesions in FPH could offer significant clinical benefits.

## Data availability statement

The original contributions presented in the study are included in the article/supplementary material, further inquiries can be directed to the corresponding author.

## Ethics statement

Ethical approval was not required for the studies involving animals in accordance with the local legislation and institutional requirements because it was obtained through an informed consent form (ICF) from the legal guardian the authorization for the publication or disclosure of the patient’s clinical data and images. Written informed consent was obtained from the owners for the participation of their animals in this study.

## Author contributions

BS: Conceptualization, Investigation, Methodology, Writing – original draft, Writing – review & editing. PT: Writing – original draft, Writing – review & editing, Conceptualization, Methodology. PB: Writing – original draft, Investigation. IA: Writing – original draft, Investigation. GL: Investigation, Writing – original draft. KN: Writing – original draft, Methodology, Resources, Validation. AG: Methodology, Writing – original draft, Conceptualization, Supervision, Writing – review & editing. PP: Investigation, Methodology, Project administration, Supervision, Writing – original draft, Writing – review & editing, Resources, Validation. RH: Investigation, Methodology, Project administration, Supervision, Writing – original draft, Writing – review & editing.
